# 1125. Effect of intravenous pulses of methylprednisolone 250 mg versus dexamethasone 6 mg in hospitalized adults with severe COVID-19 pneumonia: an open-label randomized trial.

**DOI:** 10.1093/ofid/ofac492.964

**Published:** 2022-12-15

**Authors:** Carlos Dueñas Gutierrez, Jesica Abadia-Otero, Ivan Cusacovich, Jose Ignacio Martín-González, Alberto Muela-Molinero, Luis Corral-Gudino, Roberto González-Fuentes, Ängela Ruíz de Temiño, Elena Tapia Moral, Francisca Cuadrado-Medina, Miguel Martín-Asenjo, Pablo Miramontes Gonzalez, Jose Luis Delgado Morales, Sandra Ines, Laura Abad-Manteca, Iciar Usategui-Martín, Tomás Ruíz-Albí, Sara Miranda-Riaño, Patricia Rodríguez-Fortúnez, Consuelo Rodríguez-Jiménez, Esperanza López-Franco, Miguel Marcos

**Affiliations:** Hospital Clínico de Valladolid, Valladolid, Castilla y Leon, Spain; Hospital Rio Hortega, Valladolid, Castilla y Leon, Spain; HCUV, Valladolid, Castilla y Leon, Spain; HUSAL, Salamanca, Castilla y Leon, Spain; CAULE, León, Castilla y Leon, Spain; HURH, Valladolid, Castilla y Leon, Spain; HCUV, Valladolid, Castilla y Leon, Spain; HURH, Valladolid, Castilla y Leon, Spain; HCUV, Valladolid, Castilla y Leon, Spain; HURH, Valladolid, Castilla y Leon, Spain; HCUV, Valladolid, Castilla y Leon, Spain; HURH, Valladolid, Castilla y Leon, Spain; HCUV, Valladolid, Castilla y Leon, Spain; HUSAL, Salamanca, Castilla y Leon, Spain; HURH, Valladolid, Castilla y Leon, Spain; HCUV, Valladolid, Castilla y Leon, Spain; HURH, Valladolid, Castilla y Leon, Spain; HCUV, Valladolid, Castilla y Leon, Spain; HUSAL, Salamanca, Castilla y Leon, Spain; HUSAL, Salamanca, Castilla y Leon, Spain; HUSAL, Salamanca, Castilla y Leon, Spain; HUSAL, Salamanca, Castilla y Leon, Spain

## Abstract

**Background:**

Pulse glucocorticoid therapy is used in COVID-19 infection. We evaluated the

effectiveness of methylprednisolone 250 mg/d for 3 days vs. dexamethasone 6 mg/d for 10 days in patients with severe but not critical COVID-19 pneumonia.

**Methods:**

A multicentre, randomized, open-label, controlled trial was conducted between February 2021 and August 2021 at 4 hospitals in Spain and included 128 hospitalized adults with confirmed COVID-19 pneumonia needing oxygen therapy but not critically ill. Patients were randomly assigned in a 1:1 ratio to receive dexamethasone 6 once daily for 10 days or methylprednisolone 250 mg once daily for 3 days. The primary outcome was 28-day mortality.

**Results:**

Of the 128 randomized patients, 125 were analysed (mean age 60 ± 17 years; 82 males [66%]). Mortality at 28 days was 4.8% in the 250 mg methylprednisolone group vs. 4.8 % in the 6 mg dexamethasone group (absolute risk difference, 0.1% [95% CI, -8.8 to 9.1%]; *P*=0.98). The post-hoc added composite outcome of mortality at 90 days or intubation was 15.9% in the 250 mg methylprednisolone group vs. 15% in the 6 mg dexamethasone group (absolute risk difference, -0.9% [95% CI, -13.8 to 12.3%];*P*=0.83). Hyperglycaemia was more frequent in the methylprednisolone group, at 27.0 vs. 8.1 % (absolute risk difference, -18.9% [95% CI, -31.8 to - 5.6%]; *P*=0.007).

**Conclusion:**

Among severe but not critical patients with COVID-19, 250 mg/d for 3 days of methylprednisolone compared with 6 mg/d for 10 days of dexamethasone did not result in a decrease in mortality or intubation.

**Disclosures:**

**Carlos Dueñas Gutierrez, MD**, ViIV, GILEAD, Jannsen, MSD: Lecture fees.

